# Screening, brief intervention, and referral to treatment: promoting healthy pregnancies by identifying and addressing alcohol use

**DOI:** 10.1186/1940-0640-8-S1-A55

**Published:** 2013-09-04

**Authors:** Brie Reimann, Leigh Fischer, Carolyn Swenson

**Affiliations:** 1 Peer Assistance Services, Inc. Denver, Colorado, USA; 2 HealthTeamWorks, Lakewood, Colorado, USA

## 

Sexually active women who drink and who do not use effective methods of contraceptives are at-risk of alcohol exposed pregnancies. Routinely screening and intervening for alcohol use provides an opportunity to educate women about the risks of drinking and the importance of using effective methods of contraceptives. Despite the evidence, screening, brief intervention, referral treatment (SBIRT) protocols have not been widely adopted in healthcare settings in the United States. For the past several years, Colorado has worked to integrate SBIRT as a standard of care in a variety of healthcare settings. Recently the SBIRT Colorado program expanded its approach to better identify and intervene for risky alcohol use among women of childbearing age and pregnant women, with the goal to prevent alcohol-exposed pregnancies. The SBIRT Colorado approach to identifying and intervening for risky alcohol use among women of childbearing age and pregnant women focuses on administering screening questions to assess for risky alcohol use and contraceptive use. Additionally, questions are administered to assess readiness to change alcohol use behavior and/or use of contraceptives. Brief interventions focus on providing women information about the health risks associated with risky alcohol consumption and about the harm associated with alcohol consumption during pregnancy. Data collected to date indicate that of women reporting risky alcohol behavior as indicated on the Alcohol, Smoking, and Substance, Involvement Screening Test, a high percentage report ineffective use of contraceptives. Routinely screening women of childbearing age and pregnant women and offering brief interventions provides an opportunity to prevent alcohol-exposed pregnancies.

